# Impact of high-fat diet and hypoxia on the serum levels of main vasoconstrictors in male rabbits

**DOI:** 10.15171/jcvtr.2017.15

**Published:** 2017-05-29

**Authors:** Ali Aliyev, Magid Seyedghodraty, Mustafa Mohammadi, Fariba Mirzaei, Marzieh Marahem

**Affiliations:** ^1^Department of Physiology, Baku State University, Baku, Azerbaijan; ^2^Department of Physiology, Tabriz University of Medical Sciences, Tabriz, Iran

**Keywords:** Atherosclerosis, Hypoxia, Renin, Angiotensin, Epinephrine, Norepinephrine

## Abstract

***Introduction:*** During atherosclerosis process, vasoconstriction phenomenon occurs which in turn leads to tissue hypoxia. A few studies have been performed on the combination of atherosclerosis and hypoxia as stressors that may accelerate secretion of constrictors. The aim of present study was to evaluate the effects of atherosclerosis and hypoxia on serum levels of main vasoconstrictors (epinephrine, norepinephrine and renin).

***Methods:*** In this interventional study, 32 New Zealand white rabbits were randomly divided into four groups (n = 8): normal diet (control group), normal diet exposed to hypoxia (11%, 10 days), high-fat diet (cholesterol-2%, 8 weeks), and high-fat diet with hypoxia. Later, serum levels of renin, epinephrine and norepinephrine were measured on second, 56th and 66th days.

***Results:*** High-fat diet and hypoxia caused significant increase in epinephrine and norepinephrine concentrations on days 56 and 66 compared to the control group (*P *< 0.05). Also, renin showed significance increase in high-fat diet and high-fat diet+ hypoxia groups compared to the control group (*P *< 0.05).

***Conclusion:*** Both high-fat diet and hypoxia increase renin levels in male rabbits. Furthermore, the combination of high-fat diet and hypoxia immensely increases renin levels. Both hypoxia and combined of high-fat diet and hypoxia increase norepinephrine levels. However epinephrine is only increased in the combination of high-fat diet and hypoxia. So the presence of hypoxia in combination with high-fat diet, cause accelerated and aggravated atherosclerosis.

## Introduction

Atherosclerosis is the leading cause of mortality in the developed world. Atherosclerosis syndrome affecting arterial blood vessels. This leads to the development of cardiovascular disease including myocardial infarction (MI) and stroke.^1^ The characteristic lesion of atherosclerosis is the fibrous plaque composed mainly the accumulation of endothelial cells skeletal muscle cells, T type lymphocytes and macrophages.^2,3^ Atherosclerosis begins with fatty streak which is a accumulation of lipid in the intima layer of the artery. Fatty streak evolve to fibrous plaque and unstable plaque.^3^ Vasoconstrictors including epinephrine, norepinephrine and renin-angiotensin are the key mechanical forces that affect the blood flow and stability of atherosclerotic plaques.^4^ Activation of the renin-angiotensin system has different effects on cardiovascular system activity such as vasoconstriction and release of thromboxane A2.^5^ Blood vasoconstrictors, in long term, can cause high blood pressure, cardiac and vascular hypertrophy and progression of atherosclerotic plaques.^2^ Some Studies have demonstrated that treatment with inhibitors of angiotensin-converting enzyme (ACEI) significantly reduced the incidence of myocardial attacks.^6,7^ Laboratory studies, similar to experimental findings, have shown that use of angiotensin antagonists, reduced the risk of atherosclerosis.^8^ Some researchers have suggested that angiotensin has a direct effect on vascular wall through an intracellular mechanism.^9^ Unlike the known effects of angiotensin in atherosclerosis development, effects of catecholamines are different in ischemic and normal conditions.^10,11^ Activity of vasoconstrictor system increases following hypoxia in order to increase the cardiac output and compensate the oxygen deficiency.^12^ It was reported that elevated levels of catecholamines attributed to the hypertension in some conditions such as sleep apnea.^13^

Studies have shown that atherosclerosis is a process in which vasoconstriction occurs; this in turn leads to tissue hypoxia. Combined atherosclerosis and hypoxia may intensify the secretion of vasoconstrictors and further harm the oxygen supply to tissues. The present study was conducted to evaluate the effects of atherosclerosis and hypoxia on blood content of vasoconstrictors levels of epinephrine, norepinephrine and renin.

## Material and Methods

Thirty-two male New Zealand white rabbits were obtained from Pasteur Institute, Iran with an average weight of 2.05 ± 0.15 kg. They were held in animal room in pharmaceutical research center with laboratory temperature of 20 ± 2°C. Animals were randomly divided into four groups (n = 7): 1) control (C), 2) hypoxia (H), that animals exposed to O_2_ 11% for 10 days. 3) Atherogenic diet (A): This group were fed a diet contain 2% cholesterol for 8 weeks. 4) Atherogenic diet + hypoxia (A+H); this group received high- fat diet for 8 weeks and was exposed to hypoxia 11% in first ten days of diet. Cholesterol powder (Merck Co, USA) was mixed in to the feed (2%) The animals in hypoxia groups were maintained in GO2 Altitude hypoxia chambers (O_2_ 11%) manufactured by Biomedtech (Australia). Blood samples were collected from left ear at 10 am on second, 56th and 66th days. The samples were centrifuged in 3500 rpm for 15 minutes; then, obtained serums were stored at -80°C until analyze time. The levels of renin (LSBIO Rabbit ELISA kit), epinephrine and norepinephrine were measured with special kits (Abnova kA1861).

### Statistical analysis

All data were expressed as mean±SEM. Results were compared with each other using one-way analysis of variance (ANOVA) with Tukey post hoc tests. *P* ≤ 0.05 was considered statistically significant.

## Results

### Effect of hypoxia and high-fat diet on lipid profile

Our results clearly demonstrated that 8 weeks consumption of 2% cholesterol diet significantly increased serum total cholesterol, high-density lipoprotein (HDL), low-density lipoprotein (LDL), and TG. Table 1 shows that high-fat diet with hypoxia could significantly decrease these parameters and lipid profile were enhanced in H and A+H groups in comparison with other groups significantly (P<0.001; Table 1).

**Table 1:** Comparison of serum lipid profile (in mg/dL) among 4 groups

		** TC**	** LDL**	** HDL**	** TG**
**Control**	Day 2	47.03±0.3	7.23±0.4	15.07±0.4	44.04±1.3
	Day 56	48.51±0.8	8.51±1.5	16.5±1.5	98.51±0.4
	Day 66	49.91±0.7	8.51±1.2	16.02±1.2	89.91±1.7
**Hypoxia**	Day 2	47.07±1.3	14.02±0.4*	16.02±1.4	71.04±1.4*
	Day 56	49.71±0.7	15.5±1.5*	16.5±1.7	70.5±1.6*
	Day 66	51.61±0.4	15.02±1.2*	17.02±1.7	76.02±1.26
**Atherogenic**	Day 2	457.07±1.3*	655.07±7.3*#	57.03±2.3*#	64.31±2.4*
	Day 56	649.71±0.27*#	549.03±5.27*#	54.51±3.8*#	389.2±12.7*#
	Day 66	851.61±1.47*#	651.61±8.1*#	49.71±4.7*#	581.04±14.4*#
**Atherogenic and Hypoxia**	Day 2	547.07±1.43*#	655.07±7.3*#	54.03±2.2*#	68.01±2.4*#
	Day 56	859.71±0.32*#$	749.05±5.27*#$	59.31±3.8*#	459.2±15.7*#
	Day 66	984.61±0.98*#$	851.71±8.1*#$	62.51±5.7*#$	751.04±18.4*#$

Effect of hypoxia and high-fat diet on lipid profile. Data are expressed as mean ± SEM for 7 animals.* *P* < 0.05 vs the control group, ^#^*P* < 0.05 vs the hypoxia, ^$^*P* < 0.05 vs the atherogenic group in same days (n = 7).

### Effect of hypoxia and high-fat diet on renin

**Figure 1:**
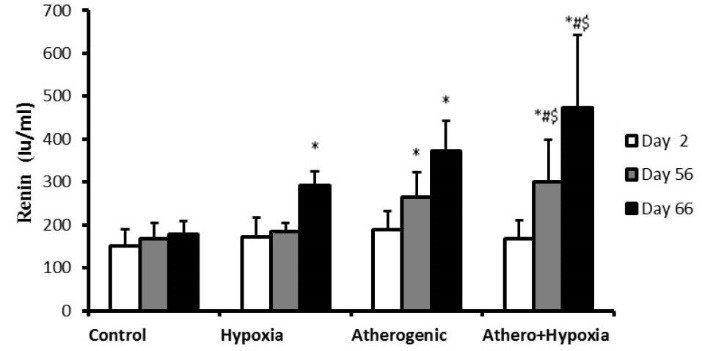
Effect of hypoxia (11%, 10 days) and high-fat diet (2% cholesterol, 8 weeks) on renin. Data are expressed as mean ± SEM for 7 animals.* *P* < 0.05 vs the control group, #*P* < 0.05 vs the hypoxia. $*P* < 0.05 vs the atherogenic group in same days (n = 7).

Our result showed that there was not statistically significant difference between groups on second day. However, on 56th day, levels of renin increased in A and A+H groups compared to the control groups (*P* <0.001). On 66th day, renin concentration increased in all groups compare to the control group significantly (*P* <0.001). Also, renin levels were enhanced in A+H group in comparison with A and H groups on not only 56th but also 66th day significantly (*P*<0.001; Figure 1).

### Effect of hypoxia and high-fat diet on epinephrine

**Figure 2:**
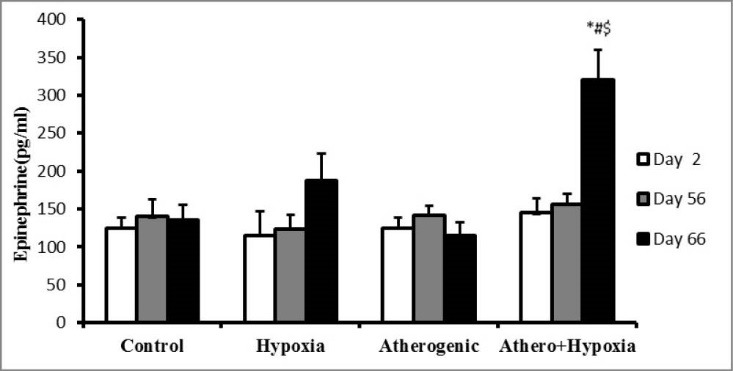
Effect of hypoxia (11%, 10 days) and high-fat diet (2%cholesterol, 8 weeks) on epinephrine. Data are expressed as mean ± SEM for 7 animals.* *P* < 0.05 vs the control group, #*P* < 0.05 vs the hypoxia. $*P* < 0.05 vs the atherogenic group in same days (n = 7).

There was not any significant difference both on days 2 and 56 between groups. However, on 66th day, but epinephrine increased in A+H group versus other groups significantly (*P* <0.001; Figure 2).

### Effect of hypoxia and high-fat diet on norepinephrine

**Figure 3:**
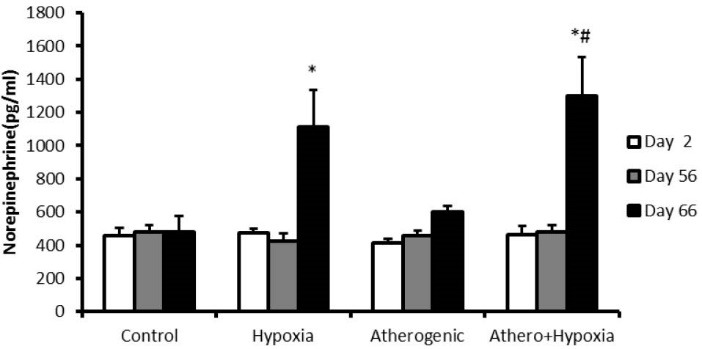
Effect of hypoxia (11%, 10 days) and high-fat diet (2% cholesterol, 8 weeks) on norepinephrine. Data are expressed as mean ± SEM for 7 animals.* *P* < 0.05 vs the control group, # *P* < 0.05 vs the hypoxia in same days (n = 7).

Results showed that there was not any difference between groups on both second and 56th days. But on 66th day, norepinephrine increased in H and A+H groups in comparison to control group significantly (*P* <0.001).Also, there was significant different between A+H and H groups (*P* <0.001; Figure 3).

## Discussion

Our results indicated that 8 weeks consumption of 2% high-cholesterol diet increased all serum cholesterol profile fractions and induced formation of atherosclerotic lesions. Moreover, Results obtained from our study showed that renin concentrations increased during chronic hypoxia and high-fat diet; this increase was less for hypoxia compared to atherosclerosis. During hypoxic-atherosclerosis condition, the increase in renin concentration was higher than each conditions alone, which explains the synergistic effects of these two conditions. It should be noted, a study showed that high-fat diet (2% cholesterol) can create atherosclerosis in arteries such as aorta, carotid and renal arteries.^14^ In this regard, Krämer et al showed that in rats exposed to hypoxia condition (8%) for 6 hours, plasma renin activity RAS increased up to four-fold; furthermore, the levels of renin mRNA increased^15^ which is consistent with our results. Czyzyk‐Krzeska and Trzebski^16^ and Malpas et al showed that the activity of renal nerves increased during hypoxia, which is likely to induce renin secretion.^16,17^ Regarding increased renin secretion during atherosclerosis, it should be mentioned that the relationship between RAS and atherosclerosis has been revealed in many publications. Nevertheless, whether atherosclerosis itself leads to increased renin secretion and triggers renin–angiotensin system is yet to be elaborated. Our research indicates that the activation of RAS worsens the atherosclerosis condition and activates the RAS system through a positive feedback. Comparing our results obtained from atherosclerosis and atherosclerosis-hypoxia conditions on day 66, and considering the significant changes between two groups, it might be said that hypoxia condition promotes the secretion of renin. In our study, the increase in norepinephrine concentrations was 2 times higher than epinephrine concentration in hypoxia group, while this increase in group A was about 1.5 times compared to group A+H. So, in both groups, norepinephrine showed a further rise. Surks et al and Sharma et al emphasized on increased activity of sympathetic system in chronic hypoxia.^18,19^ There is no consistency about the origins of circulating catecholamines, and some considered the central part of adrenal responsible for this increase. Johnson et al suggested the activation of sympathetic and adrenal systems during moderate to severe hypoxia.^20^ Other research showed that the severity and duration of hypoxia have different effects on each of these mechanisms. Severe and short term hypoxia increases the activity of sympathetic system; while, moderate hypoxia suppresses the activity of the sympathetic system.^12^ Response of central part of adrenal is seen in all intensities of hypoxia.^21^ It is also demonstrated that hypoxia at a concentration of 10.5% oxygen for 6 hours, increases the activity of adrenal central part by 40%‘while, acute severe hypoxia leads to a sharp rise in activity of adrenal central part up to 10 fold. Chronic hypoxia with moderate intensity (11%) showed a 2-fold increase in sympathetic nervous activity during 14 days, while these results were not observed on early days. This represents a pivotal role of adrenal central part in activating sympathetic system.^9^ Although there is no known mechanism to relate central part of adrenal to hypoxia, some publications pointed that the central part of the adrenal is stimulated by hypoxia which subsequently increases the blood catecholamines.^22^ A study performed on dogs by Favier et al showed that norepinephrine increased during hypoxia, but there was no increase in epinephrine levels,^23^ which was different with our results for epinephrine. Hammill et al also showed that removal of adrenal effectively reduced the effects of catechol amines during exercise compared to blocking sympathetic nervous system.^21^ Similar to our results, the epinephrine and norepinephrine enhancement was observed during chronic hypoxia.^24^ It was showed that epinephrine deteriorated the lesions of atherosclerosis in monkeys.^21^ Also it was seen that the MI occurred in monkeys fed by cholesterol-rich diet subsequent to norepinephrine infusion.^25^ In addition to catecholamines role in vasoconstriction, it has been shown that they can deteriorate atherosclerosis lesions in animals and humans explaining their involvement in the pathogenesis of atherosclerosis. Bauch et al showed that epinephrine and norepinephrine stimulated proliferation of cultured endothelial cells and smooth muscle cells in rat and pig aorta. Also atherosclerotic risk factors increased catecholamines levels. Determination of plasma epinephrine and norepinephrine concentration in patients suffering from diabetes and coronary artery disease showed that in coronary artery disease, epinephrine and norepinephrine levels were higher than diabetic patients.^26^ Then we can suggest that catecholamines may play a role in the development and subsequent complications of atherosclerosis.

### Clinical implications and limitations of the study

The model used in this study was similar to COPD model, so we can generalize to these patients. In Limitations of the Study we had financial problems and could not provided histological images.

## Conclusion

Hypoxia increased renin, epinephrine and norepinephrine levels. Also addition of high-fat diet to hypoxia increased these hormones significantly. Nevertheless, high-fat diet alone only increased renin levels significantly and had no significant impact on epinephrine and norepinephrine levels.

## Competing interests

The authors declare that they have no competing interests.

## Ethical Approval

All experiments were designed to minimize the number of rabbit used and carried out via law which was in complete compliance with the *Guide for the Care and Use of Laboratory Animals,* published by National Academies Press.

## Acknowledgments

This study was financially supported by physiology department of faculty of medicine at Tabriz University of Medical Sciences.
